# 

**Published:** 1873-05

**Authors:** 


### Contents of May Number.

### ORIGINAL DEPARTMENT.

ART. I.—Proceedings of the Albany County Medical Society. Reported by F. C. Curtis, M. D. .................... 361
ART. II.—Varicose Ulcers; Their Causation, Pathology and Treatment. By C. C. F. Gay, M. D. .................... 375
ART. III.—Rubber Bands in the Treatment of Fractures. By J. W. Southworth, M. D. .................... 380
ART. IV.—Rubber Bandage. By U. C. Lynde, M. D. .................... 383

### MISCELLANEOUS.

Thoracentesis Remarks upon twenty cases. By Austin Flint, M. D ..... 384
Clinical Remarks upon the Treatment of Syphilis. By Willard Parker, M. D .................... 386
Functional Dyspepsia, Anæmia, and Chlorosis, New Treatment of. By C. E. Brown-Sequard, M. D. .................... 388
Pancreatic Emulsion in Wasting Diseases of Children .................... 391
Replantation of Teeth .................... 393

### EDITORIAL DEPARTMENT.

Twenty-fourth Annual Meeting of the American Medical Association ... 393
Obituary Notice of the death of Dr. Hiram Taber .................... 396
Erie County Medical Society Meeting .................... 397
Medical Books of Lindsay & Blakiston .................... 397

BOOKS REVIEWED:

A Handbook of Post Mortem Examinations and of Morbid Anatomy. By Francis Delafield, M. D .................... 397
Diseases of the Urinary Organs. By John W. S. Gouley, M. D .................... 398

Books and Pamphlets Received .................... 400

